# Relationship between sucrose concentration and bacteria proportion in a multispecies biofilm: **Short title**: Sucrose challenges to a multispecies biofilm

**DOI:** 10.1080/20002297.2021.1910443

**Published:** 2021-04-07

**Authors:** Jian-Na Cai, Hyeon-Mi Choi, Jae-Gyu Jeon

**Affiliations:** a Department of Oral Biology, Binzhou Medical University, Yantai, People’s Republic of China; b Department of Dentistry, Presbyterian Medical Center, Jeonju, Republic of Korea; c Department of Preventive Dentistry, School of Dentistry, BK21 Plus Program, Jeonbuk National University, Jeonju, Republic of Korea

**Keywords:** Sucrose, multispecies biofilm, dental caries, relationship, microbial homeostasis

## Abstract

**Objective**: The aim of this study was to evaluate the relationship between sucrose concentration and bacteria proportion in a multispecies biofilm model. **Methods**: *Streptococcus mutans* (*S. mutans), Streptococcus oralis* (*S. oralis*), and *Actinomyces naeslundii* (*A. na*es*lundii*) were chose to form a multispecies biofilm. Different concentration (0–40%) of sucrose was introduced to the multispecies biofilm 3 times per day (30 min per time). And then the bacteria proportion and acid production of the biofilms were analyzed. **Results**: Increasing sucrose level increased CFU count of *S. mutans* up to a certain concentration (5% sucrose), after which the number of *S. mutans* slightly decreased, but the CFU counts of *S. oralis* and *A. na*es*lundii* continually decreased with sucrose concentration increase, especially, from 5% sucrose, the reduction was significant, and *S. mutans* became the dominant species in the biofilms. Furthermore, the acid production ability of the multispecies biofilm gradually increased and slightly decreased with sucrose concentration increased, and the turning concentration was 5%. **Conclusion**: Our findings suggest that increasing sucrose level could increase the competitiveness of *S. mutans* in the multispecies biofilm, which may shift the biofilm to a more cariogenic one, and 5% sucrose formed a most cariogenic biofilm in this study.

## Introduction

Dental biofilm, which is closely related to dental caries, is composed of diverse and complex oral microorganisms. It is estimated that more than 700 different types of microorganism have been isolated from dental plaque [1]. The composition of the resident microflora remains stable over time, which termed microbial homeostasis. However, a change in a key environmental factor will breakdown this microbial homeostasis, such as the low pH condition generated from dietary carbohydrate metabolism, can promote the outgrowth of mutans streptococci and lactobacilli and decrease the level of *S. sanguinis* and other oral streptococci [1,2]. This shift in microbial composition to aciduric and acidogenic bacteria will increase the amount of acid production and lead to the demineralization of teeth, which will increase the risk of caries formation.

Among dietary carbohydrates, sucrose is generally regarded as one of the most cariogenic carbohydrate, because it is fermentable and also serves as a substrate for the synthesis of polysaccharides, especially extracellular polysaccharides (EPSs) in dental plaque, which play important role in caries development [3–5]. To date, a large amount of epidemiological and experimental studies have demonstrated a positive response relationship between sucrose and dental caries. Hefti and Schmid [6] demonstrated that higher sucrose intake could increase the incidence of smooth surface and fissure caries in an animal study. While other studies have shown that the prevalence of dental caries derived from the consumption of sucrose is dependent on the patterns of sucrose intake, including physical presentation, quantity, and frequency [7,8]. Furthermore, studies in Japanese children during World War II reported a positive linear relationship between the log of annual caries incidence rate and national sugars consumption per capita per year [9,10]. And a longitudinal study among Finnish adults suggests a linear dose–response relationship between sugars and caries, with amount of intake being more important than frequency of ingestion [11].

Our previous studies have demonstrated that long- or short-term sucrose treatment can affect *S. mutans* biofilm formation in a second-order polynomial curve pattern with concentration dependence [12,13]. However, these studies all performed experiment with mono-species biofilm, which cannot simulate the complex microflora in dental biofilms. Therefore, in this study, we selected *S. oralis*, one of the most commonly detected pioneer colonizers in dental biofilm, *A. naeslundii*, which is also detected during the early stages of plaque formation and may be associated with development of root caries, and *S. mutans*, one of the most cariogenic bacteria [4,14] to form a multispecies biofilm to mimic the diverse bacteria community in dental biofilm to some extent. With this multispecies biofilm model, we evaluate the relationship between sucrose concentration and bacteria proportion.

## Materials and methods

### Multispecies biofilm formation and experimental scheme

**Figure 1.**
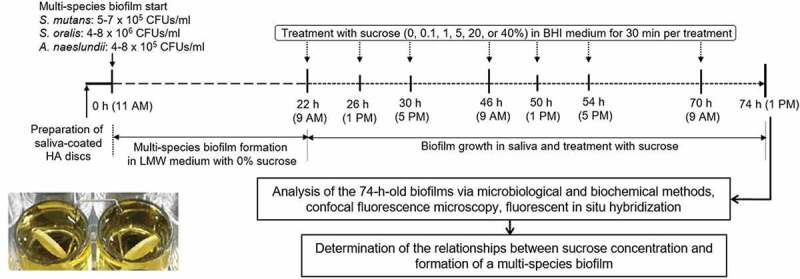
Formation of multispecies biofilm and the experimental schemes of this study

The multispecies biofilm preparation and experimental schemes for this study are shown in Figure 1. *S. mutans* UA159, *S. oralis* ATCC 35,037 and *A. naeslundii* KCTC 9013 were used in the present study. Multispecies biofilms were formed on saliva-coated hydroxyapatite (sHA) discs (2.93 cm^2^; Clarkson Chromatography Products, Inc., South Williamsport, PA, USA) placed in a vertical position in 24-well plates using a disc holder. Briefly, HA discs were incubated in filter-sterilized (0.22-μm low protein-binding filter) human saliva for 1 h at 37°C. For biofilm formation, the sHA discs were transferred to a 24-well plate containing 0% sucrose (w/v) ultrafiltered (10 kDa molecular weight) tryptone yeast-extract (UTE) broth with *S. mutans* (5–7 × 10^5^ colony forming units (CFUs)/ml), *S. oralis* (4–8 × 10^6^ CFUs/ml) and *A. naeslundii* (4–8 × 10^5^ CFUs/ml) (2.8 ml/disc), which mimic the bacteria proportion in saliva of normal oral cavity. The biofilms were grown undisturbed at 37°C with 5% CO_2_ for 22 h to allow initial biofilm growth. After 22 h, the biofilms were grown in filter-sterilized human saliva diluted with adsorption buffer (1:1) to the end of the experimental period (74 h). The biofilms in diluted saliva were treated three times per day (at 22, 26, 30, 46, 50, 54, and 70 h) with 0, 0.1, 1, 5, 20, or 40% sucrose in brain heart infusion (BHI; Difco, Detroit, MI, USA) broth. Specifically, the biofilms were exposed to the treatments for 30 min, dip-washed with water, and then transferred to diluted saliva. Finally, the 74 h-old treated biofilms were analyzed in this study.

### Determination of bacterial count in multispecies biofilm

The number of CFUs of the sucrose treated biofilms were determined as described elsewhere [15]. Briefly, the treated biofilms were transferred into 2 ml of 0.89% NaCl and sonicated in an ultrasonic bath (Power sonic 410; Hwashin Technology Co., Seoul, Korea) to disperse the biofilms. The suspension was homogenized by sonication (VCX 130PB; Sonics and Materials Inc., Newtown, CT, USA) for 30 s after adding 3 ml of 0.89% NaCl. An aliquot (100 μl) of the homogenized suspension was serially diluted and plated to determine the number of CFUs. Especially, Mitis-salivarius agar plus bacitracin (100 μl/L) and sucrose (10%) (MSB) for quantification *S. mutan*s [16], cadmium sulfate fluoride acridine trypticase (CFAT) agar for quantification *A. naeslundii* [17], and BHI agar with 5% defibrinated sheep blood was used for *S. oralis* [18]. Differentiation of *S. oralis* in blood agar was achieved by observation of colonial morphology.

### pH drop assay

To evaluate differences in acid production of the sucrose treated multispecies biofilms, a biofilm pH drop assay was performed as described elsewhere [19]. Briefly, the treated biofilms were incubated in 20 mM PPB/1 mM MgCl_2_ + 50 mM KCl (pH 7.2) for 1 h to deplete endogenous catabolites and then transferred to a 12-well plate containing a salt solution (50 mM KCl + 1 mM MgCl_2_, pH 7.0). The pH was adjusted to 7.2 with 0.2 M KOH solution. Glucose was then added to the mixture to produce a final concentration of 1% (w/v). The pH change was assessed using a glass electrode over a period of 240 min (Futura Micro Combination pH electrode, 5 mm diameter; Beckman Coulter Inc., CA, USA).

### Confocal laser scanning microscope (CLSM) analysis

To investigate the effect of sucrose concentration (0, 1, 5 and 40%) on bacteria proportion and morphology in multispecies biofilm, fluorescence *in situ* hybridization (FISH) was performed according to previous studies [20–22]. Briefly, the oligonucleotide probe 5′-ACTCCAGACTTTCCTGAC-3′ labeled with Cy3 was used to detect *S. mutans* [23]. The oligonucleotide probe 5′-CTACACACGTGCTACAATGG CT- 3′ labeled with Cy5 was used to detect *S. oralis* [24]. The oligonucleotide probe 5′-GCTACCGTCAACCCACCC-3′ labeled with 6-FAM was used to detect *A. naeslundii* [21]. The custom synthesized probes are specific to 16S rRNA of the three bacteria, which we have confirmed in the previous study (data not show). For the sample preparation, the treated biofilms on sHA discs were first incubated in 100% ethanol for 15 min, and then fixed in 4% paraformaldehyde in phosphate-buffer saline (PBS) (pH 7.2) for 12 h at 4°C. After fixation, all specimens were washed with PBS and incubated again in a solution containing 50% ethanol in PBS for 12 h. Subsequently, the specimens were incubated in a solution containing 10 mg/ml of lysozyme (1 M Tris-HCl pH 8) for 30 min at 37°C in order to permeabilize cells. And then the samples were dehydrated with a series of ethanol washes containing 50, 80 and 100% ethanol for 3 min each and dried. Then, the fixed biofilm cells were incubated with the oligonucleotide probes at a concentration of 20 ng each per 1 ml hybridization buffer (0.9 M NaCl, 20 mM Tris-HCl pH 7.2, 0.01% SDS and 25% formamide) for 120 min at 46°C. After hybridization, samples were incubated for 15 min at 48°C in wash buffer (20 mM Tris-HCl pH 7.2, 5 mM EDTA, 0.01% SDS and 159 mM NaCl). The treated biofilm cells were rinsed with ice-cold water and air dried for CLSM.

To evaluate the relationship between sucrose concentration and bacteria viability in multispecies biofilm, the sucrose treated biofilms were stained at room temperature in the dark for 30 min using the FilmTracer live/dead Biofilm viability kit L10316 (Invitrogen, Molecular Probes Inc., Eugene, OR, USA). The final concentrations of SYTO 9 and propidium iodide (PI) were 6.0 μM and 30 μM, respectively. This live/dead kit is based on plasma membrane integrity to determine live and dead cells. In this study, we regarded the cells with intact membranes (green) as the live cells, whereas cells with damaged membranes (red) were regarded as the dead cells.

The CLSM imaging was performed using the LSM 510 META (Carl Zeiss, Jena, Germany) equipped with argon-ion and helium–neon lasers. The excitation wavelengths for FAM, Cy3 and Cy5 are 488, 543 and 633 nm, respectively. And the excitation wavelengths for SYTO 9 and PI are 488 and 543 nm, respectively. Three independent experiments were performed and five image stacks per experiment were collected (*n* = 15). The bio-volume (μm^3^/μm^2^) and thickness (μm) were quantified from the entire confocal stacks using COMSTAT image-processing software [25]. The bio-volume is defined as the volume of the biomass (μm^3^) divided by the surface area of the substratum (HA discs) (μm^2^). The three-dimensional architecture of the biofilms was visualized using Imaris 8.0.2 (Bitplane, Zurich, Switzerland).

## Statistical analysis

All experiments were performed in duplicate, and at least three different experiments were conducted. The data are presented as mean ± standard deviation. Intergroup differences were estimated using one-way analysis of variance, followed by a post hoc multiple comparison (Tukey) test to compare multiple means. Values were considered statistically significant when the *P* value was <0.05.

## Results

### Changes in bacteria counts of the multispecies biofilm

**Figure 2.**
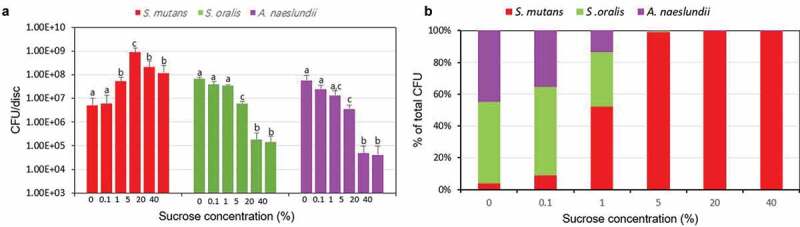
Changes in bacteria counts of the multi-species biofilm. (A) Bacterial counts. (B) Proportion of bacteria. Values in the same group (*S. mutans* group, *S. oralis* group, *A. naeslundii* group, respectively) followed by the same superscripts are not significantly different from each other (*P* > 0.05)

As shown in Figure 2A, the CFU counts of bacteria in the multispecies biofilm were changed significantly in the presence of different concentration of sucrose. The number of *S. mutans* organisms gradually increased and then decreased with sucrose concentration increased, in which 5% sucrose showed the highest CFU counts (*P* < 0.05). However, with sucrose concentration increase, the number of *S. oralis* and *A. naeslundii* appeared to decrease, especially in 20 and 40% sucrose (*P* < 0.05). To show the relationship between sucrose concentration and bacteria proportion clearly, the proportion of bacteria in multispecies biofilm was calculated. As shown in Figure 2B, there was a tendency to an increase in the proportion of *S. mutans* relative to total bacteria, whereas the proportion of *S. oralis* and *A. naeslundii* decreased, and starting from 1% sucrose, *S. mutans* became the dominant bacteria.

### Changes in fluorescence in situ hybridization study

**Figure 3.**
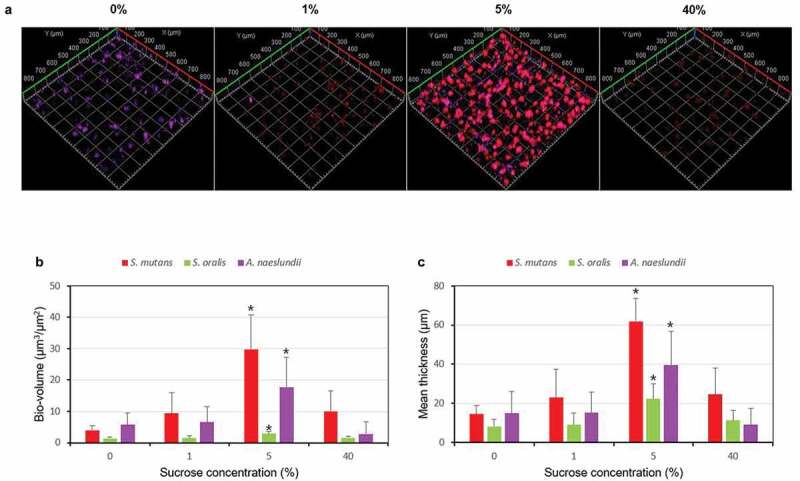
Changes in fluorescence in situ hybridization (FISH) study. (A) Representative confocal images (*S. mutans*, stained red; *S. oralis*, stained green; *A. naeslundii*, stained purple). (B) Bio-volume. (C) Mean thickness. *P < 0.05, significantly different from 0% sucrose

To further study the effect of different level of sucrose on the bacteria proportion and morphology in the multispecies biofilm, FISH study was performed, and 0, 1, 5 and 40% sucrose were chose according to the CFUs results. As shown in Figure 3A, the volume and structure of the multispecies biofilm were significantly different in different concentration of sucrose. In the presence of 0% sucrose, the biofilm showed dominance of *A. naeslundii* (purple), which was arranged in micro-colonies of varying size consisting of branching filaments, some of which were ‘spider colonies’ consisting of branching filaments radiating from a single point. However, with sucrose concentration increase, *S. mutans* (red) became the dominant bacteria, which was in a rod and regular shape. While *S. oralis* (green) was observed at all sucrose concentration scattered throughout the biofilm. These results were confirmed by the bacteria bio-volume and thickness, which gradually increased and then decreased as sucrose concentration increase, especially, the 5% sucrose, as a turning concentration, increased the bacteria volume significantly (*P* < 0.05) (Figure 3B and C).

### Changes in bacteria viability of the multispecies biofilm

**Figure 4.**
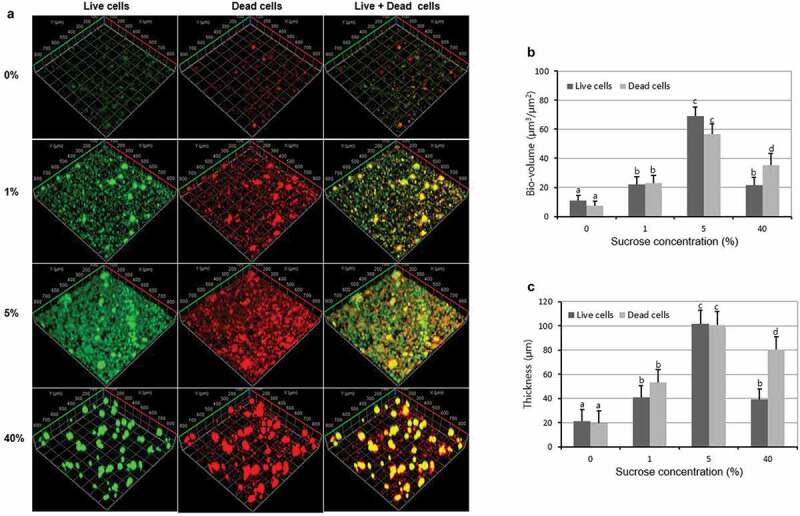
Changes in bacteria viability of the multispecies biofilm. (A) Representative confocal images (live cells, stained red; dead cells, stained green). (B) Bio-volume. (C) Mean thickness. Values followed by the same superscripts are not significantly different from each other (*P* > 0.05)

Since the FISH study only showed the total volume (live and dead) of each bacteria, therefore, live/dead biofilm viability kit was used to evaluate the effect of sucrose concentration on the bacteria viability of the multispecies biofilm. As shown in Figure 4B and C, the bio-volume and thickness of the live and dead cells gradually increased and then decreased with sucrose concentration increase, with 5% sucrose showing the highest bacterial bio-volume and mean thickness (*P* < 0.05). Furthermore, the bio-volume and thickness of live cells were similar to those of dead cells in all the tested concentration except for 40%, in which the bio-volume and thickness of dead cells were higher than those of live cells. These results were confirmed by the representative CLSM images, in which biofilms at 5% sucrose showed the highest biofilm volume and a complex structure (Figure 4A).

**Figure 5.**
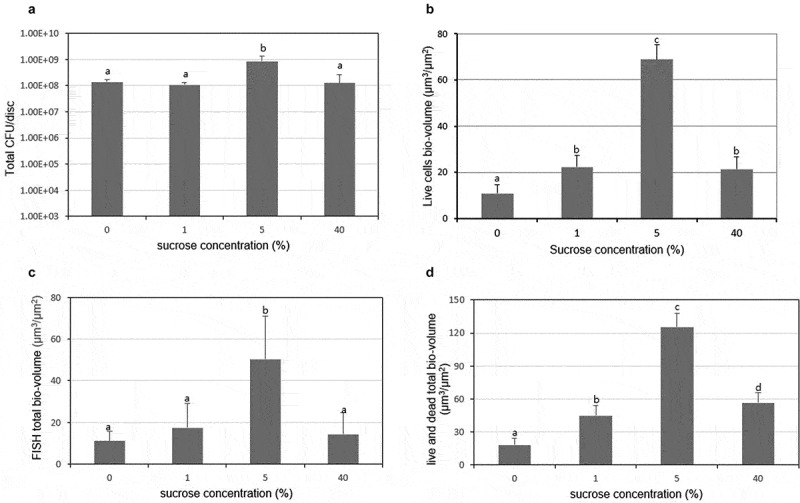
Comparison of the live and total bacteria cells in the multispecies biofilm. (A) Total bacteria counts (based on the data in Figure 2). (B) Bio-volume of live cells. (C) Bio-volume of total cells in FISH study (based on the data in Figure 3B). (D) Bio-volume of total cells in live/dead study (based on the data in Figure 4B). Values followed by the same superscripts are not significantly different from each other (*P* > 0.05)

To further validate the usefulness of the biofilm model and the reliability of the FISH method used in this study, the total CFUs (*S. mutans* + *S. oralis* + *A. naeslundii*), total FISH bio-volume (*S. mutans* + *S. oralis* + *A. naeslundii*), and total live and dead cells bio-volume were analyzed. As shown in Figure 5A and B, the total CFUs showed a similar pattern with the live cells bio-volume with sucrose concentration increase. Furthermore, the total FISH bio-volume in different concentration of sucrose followed a similar trend with those in total live and dead cells as sucrose concentration increase (Figure 5C and D). In addition, in all the results, 5% sucrose showed the highest bacteria bio-volume (*P* < 0.05).

### Changes in acid production of the multispecies biofilm

**Figure 6.**
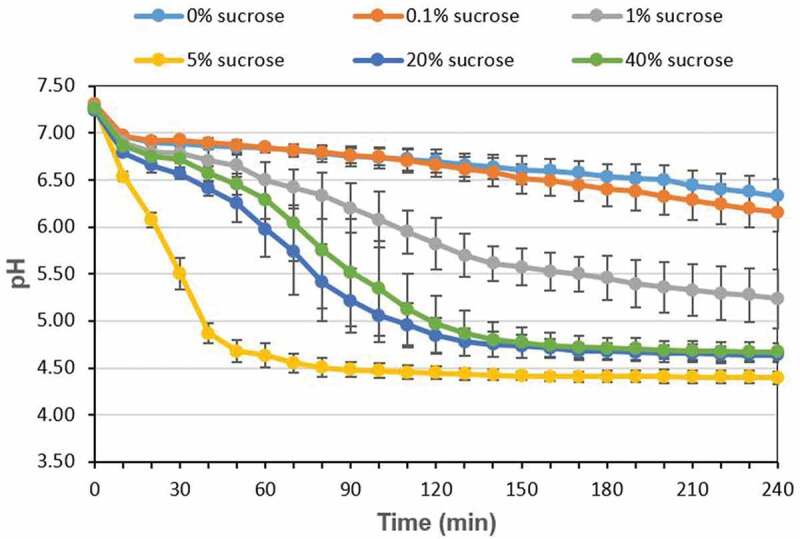
Changes in acid production of the multispecies biofilm

In addition to the changes in bacteria proportion, the acid production of the multispecies biofilm was also evaluated in different concentration of sucrose. As shown in Figure 6, in the 4 h experiment period, the pH of the multispecies biofilm gradually decreased and slightly increased as sucrose concentration increase, in which 5% sucrose showed the lowest pH number, while in the presence of 20 and 40% sucrose, the acid production decreased slightly (*P* < 0.05).

## Discussion

The ecological plaque hypothesis demonstrates that environmental perturbations, such as an increase in sucrose availability, will shift the biofilm from a dynamic equilibrium one to an aciduric and acidogenic bacteria dominated one, which will increase the risk of caries [1,2]. To further explore this conclusion more intuitively, we selected *S. mutans, A. naeslundii* and *S. oralis*, the representative bacteria for cariogenic and non-cariogenic bacteria, to form an in vitro multispecies biofilm model. With this multispecies biofilm model, we evaluate the exact relationship between sucrose concentration and bacteria proportion, especially the influence of higher level of sucrose on the proportion of cariogenic and non-cariogenic bacteria.

Although amount of in situ or in *S. mutans* single-species biofilm models have been used to evaluate the cariogenic potential of dietary carbohydrates [26–28], to demonstrate the relationship between sucrose concentration and bacteria proportion, a more complex and controlled biofilm is required, considering the differences in carbohydrate metabolism by oral bacteria and the interactions among these microorganisms. The experiment model used in this study was improved to be slightly closer to the in vivo condition. First, the three bacteria were inoculated in a specific proportion, which mimic the bacteria proportion in saliva of normal oral cavity. Second, the biofilm in free-sucrose medium was incubated for 22 h for the initial biofilm formation, and then different concentration of sucrose was introduced to the biofilm 3 times per day to imitate a human meal pattern. In addition, diluted human saliva was used to culture the biofilm for simulating the oral condition in the intervals of the three times sucrose treatment. However, although the biofilm model used in this study simulated the clinical situation to some extent, additional in vivo studies are required since dental biofilms are composed of a complex microbial community, and the ecology of the mouth is complex and dynamic, which may be influenced by the flow rate and properties of saliva, the lifestyle of an individual (in particular, the nature of the diet, and exposure to medication), and the integrity of the host defences.

Previous studies have demonstrated that increase sucrose level can shift the biofilm from a dynamic equilibrium one to a cariogenic bacteria dominated one [26,29], which is consistent with the results in this study. As shown in Figure 2, increasing sucrose level increased CFU count of *S. mutans* (cariogenic bacteria) up to a certain concentration (5% sucrose), after which the number of *S. mutans* slightly decreased, which is consistent with our previous results concluded from *S. mutans* single-species biofilm [12,13]. However, the CFU counts of *S. oralis* and *A. na*es*lundii* continually decreased with sucrose concentration increased, especially, from 5% sucrose, the reduction was significant, and *S. mutans* became the dominant species in the biofilms. These results indicate that high sucrose challenge can increase the competitiveness of *S. mutans* in mixed-species environment, although at higher sucrose concentration (20% and 40% sucrose), the CFU counts of *S. mutans* decreased slightly, it still was the dominant species in the biofilm. A possible explanation for higher sucrose concentration inhibit the bacteria viability might be related to the increase in osmotic pressure generated by higher sucrose concentration [30], which can affect biofilm bacterial growth and physiological activity such as acid production. And a previous study showed that in the presence of 20%–60% sucrose, the growth of *Listeria monocytogenes*, a gram-positive bacterium, was inhibited [31]. This also can explain the results that the bio-volume and thickness of the dead cells were higher than those of live cells at 40% sucrose (Figure 4B and C). However, the exact mechanism of osmotic pressure changes by sugars including sucrose on biofilm bacteria, especially cariogenic bacteria, has not been well defined, which need further researches.

The relationships derived from CFUs analyses were confirmed by FISH and live/dead biofilm viability studies, which showed that bio-volume and thickness of each bacteria (live and dead) (Figure 3), total bacteria (live or dead) (Figure 4) of the multispecies biofilm all gradually increased and then decreased as sucrose concentration increased (turning concentration 5%). Interestingly, the bio-volume and thickness of *S. oralis* were very low at all sucrose concentration compared to the CFUs results (Figure 2), and the morphology of *S. oralis* was small and scattered throughout the biofilm (Figure 3A). This might be related to the limitations of FISH technology, since the signal intensity could be influenced by many factors, such as growth media, fixation methods, and also the physiological activity of bacteria [32]. And in this study, the branching filaments morphology of *A. na*es*lundii* may increase its adhesion ability to withstand the fixation and washing step of FISH method compared to the round shape of *S. oralis*. However, the exact reason for the lower bio-volume and thickness of *S. oralis* in FISH results need further studies.

To further validate the reproducible of this multispecies biofilm and compare the experiment method used in this study, the results of CFU, FISH and live/dead method were analyzed. The results exhibited a similar pattern, in which the bacteria composition of the multispecies biofilm gradually increased and then decreased with sucrose concentration increased, the turning concentration was 5% (Figure 5). However, the bio-volume of live cells at 1% sucrose were significantly higher than those in the control (Figure 5B), while the total CFUs were similar between two concentrations (Figure 5A). This may be explained by the different sensitivity of CFU and live/dead methods, since the cells with intact membranes are regarded as the live cells, but the CFU results show the bacteria which can recover well on the plate. In this study, in the presence of 1% sucrose, the CFU number of *S. mutans* was higher than that in control (Figure 2A), which may reinforce the adhesion of *S. oralis* and *A. naeslundii*, including cells with intact membranes but cannot recover well on the plate. Although there were a few slight differences between different methods, the overall trend is consistent, which suggests that the multispecies biofilm used in this study was reproducible and has great potential to be used in basic research, such as evaluating the cariogenic potential of dietary carbohydrate or assessing antibacterial molecules.

In addition to bacteria proportion, the acid production of the multispecies biofilm increased and then decreased as sucrose concentration increased, and 5% sucrose showed the highest acid production (Figure 6). This might be closely related to the changes in *S. mutans* counts in different sucrose level, which was highest at 5% sucrose, followed by 20% and 40% sucrose (Figure 2), since *S. mutans* is the most acidgenicity and aciduric bacteria in this multispecies biofilm. When high concentration of sucrose (>5%) is introduced to the biofilm, *S. mutans* can produce large amount of acid and survive in this low pH condition, which can further increase its competitiveness, and lead to a decrease in the number of *S. oralis* and *A. naeslundii* (Figure 2). However, in the presence of 1% and 20% (or 40%) sucrose, the CFU counts of *S. mutans* were similar, but the pH was lower at 20% (or 40%) sucrose than that at 1% sucrose. This might be explained by the intermediary characters of *A. naeslundii* in the three-species biofilm, since the CFU counts of *A. naeslundii* at 20% (or 40%) sucrose decreased significantly compared to that at 1% sucrose, and *A. naeslundii* can produce urease, which may have a role in modulating pH in biofilm [33].

In summary, the results of the present study revealed that the bacteria proportion in this multispecies biofilm model could be affected by sucrose in a concentration-dependent pattern, of which high sucrose challenge could induce a transition from a non-virulent microbial community (high levels of *S. oralis* and *A. naeslundi*i) to a highly acidogenic and aciduric population (high levels of *S. mutans*), and the sucrose turning concentration was around 5%. The results recommend that we should decrease the amount and frequency of sucrose intake in daily life, and rinse or brush the teeth timely after taking sucrose. Although the multispecies biofilm model used in this study can provide significant benefits of establishing the reproducibility of data and reducing variance, additional studies are required to confirm the exact relationship between sucrose consumption and cariogenic biofilm formation, since the environmental conditions in the present study are different from those in the oral cavity.
